# Odor of achlorophyllous plants’ seeds drives seed‐dispersing ants

**DOI:** 10.1002/ece3.7612

**Published:** 2021-06-22

**Authors:** Mikihisa Yamada, Masaru K. Hojo, Akio Imamura

**Affiliations:** ^1^ Hokkaido University of Education Asahikawa Japan; ^2^ Department of Bioscience School of Science and Technology Kwansei Gakuin University Sanda Japan

**Keywords:** directed seed dispersal, elaiosome, myco‐heterotrophic plant, myrmecochory, seed volatiles

## Abstract

Seed dispersal by ants is an important means of migration for plants. Many myrmecochorous plants have specialized appendages in their seeds called elaiosome, which provides nutritional rewards for ants, and enable effective seed dispersal. However, some nonmyrmecochorous seeds without elaiosomes are also dispersed by ant species, suggesting the additional mechanisms other than elaiosomes for seed dispersal by ants. The seeds of the achlorophyllous and myco‐heterotrophic herbaceous plant *Monotropastrum humile* are very small without elaiosomes; we investigated whether odor of the seeds could mediate seed dispersal by ants. We performed a bioassay using seeds of *M*. *humile* and the ant *Nylanderia* *flavipes* to demonstrate ant‐mediated seed dispersal. We also analyzed the volatile odors emitted from *M*. *humile* seeds and conducted bioassays using dummy seeds coated with seed volatiles. Although elaiosomes were absent from the *M*. *humile* seeds, the ants carried the seeds to their nests. They also carried the dummy seeds coated with the seed volatile mixture to the nest and left some dummy seeds inside the nest and discarded the rest of the dummy seeds outside the nest with a bias toward specific locations, which might be conducive to germination. We concluded that, in *M*. *humile* seeds, volatile odor mixtures were sufficient to induce seed‐carrying behavior by the ants even without elaiosomes.

## Graphical abstract

Despite the lack of elaiosomes on the seeds, the ants carried them back to their nests. Furthermore, those dummy seeds coated with a mixture of volatiles were also carried away.

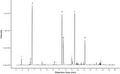

## 1 INTRODUCTION

Various seed and fruit traits, such as size and color, tend to be correlated, forming what are known as “dispersal syndromes” that may have arisen to attract particular dispersers (Brodie, 2017; Valenta & Nevo, 2020) or to facilitate transport from their parent plants via wind (anemochory) or currents (hydrochory) (Bullock & Clarke, 2000; Nathan et al., 2011; Nilsson et al., 1991; Ohnishi et al., 2008). Among the seed and fruit traits, plant secondary metabolites, such as lipids, are important factor mediating seed dispersal by birds, mammals, and insects and act as rewards and signals for these dispersers (Brodie, 2017; Valenta & Nevo, 2020).

Seed dispersal specially by ants is called myrmecochory. Myrmecochory is widespread among angiosperms and ecologically important; over 11,000 species of myrmecochorous plants in 77 families and 334 genera participate in myrmecochory across various ecosystems (Lengyel et al., 2010). Seeds of many myrmecochorous plants possess appendages, such as elaiosomes, containing various fatty acids and proteins. These are likely to be nutritional rewards for ants that disperse the seeds (Brew et al., 1989; Fischer et al., 2008; Konečná et al., 2018; Lanza et al., 1992). If the seeds are carried to an ant nest, the elaiosomes are generally consumed inside the nest before the seeds are discarded (Culver & Beattie, 1978; Fokuhl et al., 2007, 2012; Giladi, 2006). Various studies have shown that nutritional contents, such as lipids, amino acids, and proteins, in the elaiosome are important for inducing seed dispersal (Anjos et al., 2020; Fischer et al., 2008; Miller et al., 2020; Pizo & Oliveira, 2001; Sasidharan & Venkatesan, 2019). In contrast, some granivorous ants, such as species of the genera *Messor*, *Pheidole*, and *Tetramorium*, lose or abandon their nonmyrmechochorous seeds during transportation and thus disperse seeds without elaiosomes (Clemente & Whitehead, 2020; Kobayashi, 2009; Ohnishi et al., 2008, 2013; Retana et al., 2004).

Several studies indicated that seed dispersal by the ant is mediated not only by the nutritional rewards, but also by the chemical components of seeds (Pizo & Oliveira, 2001). It is revealed that nonvolatile contact chemicals, such as oleic acid, induce the seed dispersal by the ants (Pfeiffer et al., 2010; Turner & Frederickson, 2013). In contrast, the role of volatile odor signals in seed dispersal in relatively unstudied especially in seeds without elaiosomes (Nelson et al., 2019), although some volatile chemical signaling might be involved (Youngsteadt et al., 2008). Ohnishi et al. (2008) and Ohnishi et al. (2013) showed that elaiosome‐less seeds of *Chamaesyce* plants were carried by some ant species and that their seed dispersal was effective, but they have not determined the ant‐attracting chemical components of the *Chamaesyce* seeds.

One of the evolutionary significances of myrmecochory is directed seed dispersal. The advantages of the directed ant‐dispersal hypothesis are that ant nests maintain moist conditions and the seeds carried to the nest can escape desiccation and have a higher seedling survival (Anjos et al., 2020; Levey & Byrne, 1993). The ants bury the seeds at depths where humidity, temperature, and other conditions are suitable for germination (Anjos et al., 2020; Gibson, 1993). However, these are limited evidences to support the directed dispersal (Bond & Stock, 1989; Connell et al., 2016; Giladi, 2006).

**FIGURE 1:**
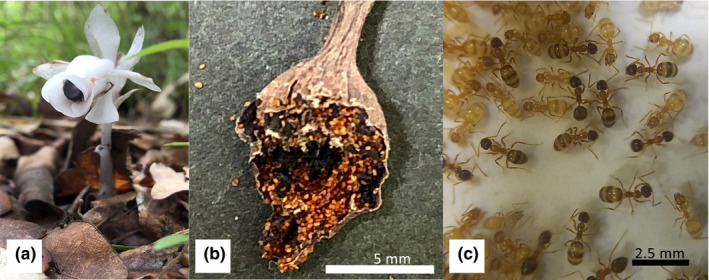
Photographs of *Monotropastrum humile* and *Nylanderia flavipes*. (a) Flower of *M*. *humile*. (b) Matured fruit and seeds of *M*. *humile*. The mature fruit of the plant is blackened and fall onto forest floor. bar: 5 mm. (c) Workers of *N*. *flavipes*. bar: 2.5 mm

*Monotropastrum humile* (D. Don) H. Hara (Ericaceae) is achlorophyllous (Figure 1)—that is, it does not have chlorophyll and does not perform photosynthesis—and inhabits forest floors in the temperate regions of Asia (Ohashi et al., 2016), which is full myco‐heterotrophic species, meaning that it parasitizes fungi throughout its life (Merckx, 2013). The plant is approximately 10 cm tall and parasitizes *Russula* and *Lactarius* species of Russulaceae (Bidartondo & Bruns, 2001), whose seeds are smaller than 0.3 mm in length and require their host fungi for germination because the seeds have only 12 cells, including the two embryos (Tanaka & Morita, 1999). Thus, seed destination is a determinant of survival. Its host, the mycorrhizal fungi of *Russula* species, inhabits the shallow areas of the forest floor (Courty et al., 2008; Rachel, 2004), and Imamura and Kurogi (2003) reported that *M*. *humile* roots inhabit the forest floor to a depth of 5–10 cm.

For seed dispersal, *M*. *humile* uses insects wandering on the ground when their berries fall to the ground during the fruiting season. Cockroaches (Uehara & Sugiura, 2017) and camel crickets (Suetsugu, 2017) have been reported as seed dispersers via endozoochory. According to these studies, *M*. *humile* berries fall to the ground during the fruiting season, and the wandering insects consume the pulpy flesh (Figure 1) around the seeds and consequently contribute to the dispersal of the seeds involved in their foraging behavior. Although *M*. *humile* seeds have no elaiosomes and seem to be nonmyrmecocohorous plants, Suetsugu (2017) and Uehara and Sugiura (2017) have reported that several ant species, including *Nylanderia flavipes* Smith (Formicidae: Formicinae), frequently visit *M*. *humile* fruits. In addition, we have observed, using a fixed‐point camera, that *N*. *flavipes* ants are attracted to the fruits and seeds of *M*. *humile* and that some of the seeds, although not many, were carried by them. Since the seeds are coated with a phlegmatic organic layer of gluey and very thin liquid, it is possible that *M*. *humile* attracts ants with fruit and seed odors and that their seeds are carried by the ants (Figure 1). The seeds might then be abandoned after the outer components are consumed (Sasidharan & Venkatesan, 2019). Consequently, the seed may be dispersed to suitable, host‐inhabited sites on the forest floor.

In this study, we investigated whether odors of *M*. *humile* seeds without elaiosomes could mediate seed dispersal by ants. Since the elaiosome‐less seeds of this achlorophyllous plant are very small and difficult to track in the field, we first performed a bioassay using intact and odorless seeds to verify whether the seed odors of *M*. *humile* are key signals for dispersal by ants. Afterward, bioassays were performed using small pieces of paper coated with the odor component of *M*. *humile* seeds as dummy seeds. We tested whether the dummy seeds were carried into the nests and were discarded to specific locations by the ants.

## 2 MATERIALS AND METHODS

### 2.1 Plant and ant materials

We used the polyphagous ant species *N*. *flavipes,* which has been shown to collect plant seeds in the field (Hosoishi et al., 2019; Tanaka & Tokuda, 2016). Ten *N*. *flavipes* colonies were collected from June to July 2019 from sites at Yuminariyama Mountain and the Hokkaido University of Education Asahikawa Campus in Hokkaido, Japan (approximately 43.79N, 142.33E). All of the colonies collected were queenless, consisting of approximately 60 workers and numerous eggs, pupae, and larvae. *Nylanderia flavipes* has polydomous colony structure and usually found as queenless in the field (Ichinose, 1986). *Monotropastrum humile* populations and *N*. *flavipes* colonies coexist sympatrically at Yuminariyama Mountain. We also collected *M*. *humile* fruits at Yuminariyama Mountain in August 2019 and stored them in a screw tube at 4°C.

The ant colonies were kept in a container that consisted of an artificial nest and a foraging arena (Figure 1). A polypropylene box (200 mm length × 136 mm width × 68 mm height) was used as the foraging arena. Fluon was applied to the upper 3 cm inside the arena to prevent ant escape. A vinyl tube (10 mm diameter × 120 mm length) was used as an artificial nest and connected to the side wall of the container at a height of 15 mm. To prevent the artificial nest from drying out, an absorbent piece of cotton was inserted at the wall side of the nest and moistened with distilled water of 5 ml every three days. The absorbent cotton was replaced every two weeks. Tuna and honey of about 0.5 g each and about 100 µl water were supplied to ants with daily replacement.

**FIGURE 2:**
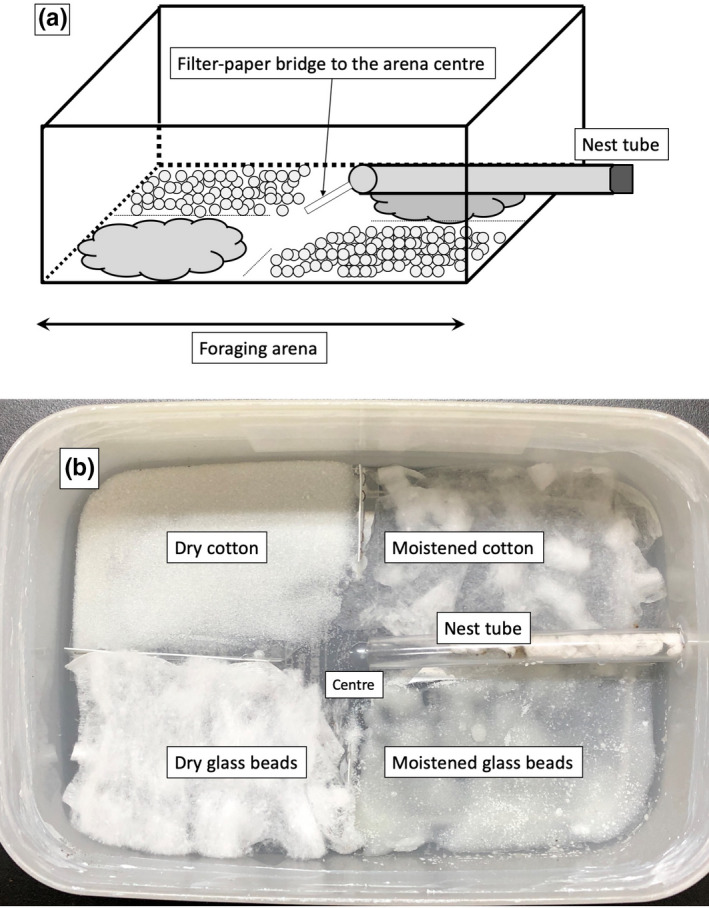
Experimental design of the ant nest and their foraging arena. (a) Nest tube is on the wall of the arena, and filter paper bridged the nest to the center of the arena. (b) Foraging arena was divided into four sections each filled with moist and dry cotton, moist and dry glass beads

The foraging arena was divided into four equivalent sections to investigate whether the ants discarded seeds in areas with different conditions. The four sections were filled with dry glass beads (1.5–2.5 mm in diameter), moistened glass beads, dry cotton wool, and moistened cotton wool. We used the beads and wool to standardize and simplify the substrate conditions, since real soil pebbles or litter, which are rather heterogeneous substrata. Glass beads simulated an environment chemically as inorganic substances, such as sand or small pebbles on the forest floor, and each section was filled with approximately 10 g of beads. Cotton wool simulated an environment chemically as organic substances, such as fallen and decomposing litter, and each section was filled with approximately 0.25 g of cotton. Since the ant species favor moist conditions to inhabit (Kallal & LaPolla, 2012), it is possible they also favor moist conditions in foraging and discarding behavior. Thus, distilled water of 5 ml was supplied to the moistened sections every three days. The entrance to the nest was placed at the center of the arena so that the four sections were equally aligned for the ants. The entrance of the nest floats was bridged with filter paper (Figure 2a). In the bioassay, the seeds or the dummy seeds were placed at the center of the arena near where the bridge connected the different sections (Figure 2b). All the containers were kept indoors at 25°C.

### 2.2 Volatile collections and chemical analyses

The volatiles emitted from *M*. *humile* seeds were collected using solid‐phase microextraction (SPME) with a 66‐µm PDMS/DVB fiber (Supelco, Bellefonte, PA, USA). The seeds with pulp were placed in 4‐ml glass vials and sealed with aluminum foil. The SPME fiber was inserted into the covered vial and volatiles were sampled for 1 hr at room temperature. Blank assays with empty vials were performed before each volatile collection session. Three independent samples were collected and analyzed.

Immediately after sampling, the SPME fiber was injected into the split/splitless injector of a gas chromatograph (GC17A; Shimadzu, Japan) equipped with a DB‐WAX column (30 m length × 0.25 mm inner diameter × 0.25 µm film thickness; J & W Scientific Inc., Folsom, CA, USA) and mass spectrometry detectors (QP5000; Shimadzu, Japan) with electron impact ionization (70 eV). The oven temperature was maintained at 40°C for 5 min, programmed to increase to 220°C at a rate of 10°C/min, and held isothermally for 10 min. The SPME fiber was desorbed in splitless mode for 1 min, while the injector and interface temperatures were at 220°C. Helium was used as the carrier gas, and the column head pressure was 100 kPa. Volatile compounds were tentatively identified by matches with the NIST mass spectral database, and identifications were confirmed by matching mass spectra and Kovat's retention index with those of commercially available authentic standards.

### 2.3 Bioassay using *M. humile* seeds

We performed bioassays using seeds of *M*. *humile,* 20 fresh untreated or solvent‐treated seeds (odorless seeds) per turn of the bioassay. For the solvent‐treated seeds, 20 fresh seeds of *M*. *humile* were soaked in ethanol (99.5%, Wako Pure Chemical Industries, Ltd.) for 30 min. Four replicate colonies resulted in a total of 80 seeds for each treatment. Untreated and solvent‐treated seeds were assayed separately to avoid odor transfer, in which the treatment order for each colony was randomized by the function “sample” of “base” package of R 4.0.3 for Mac OSX (R core team, 2018). The colonies were starved (only given water) for 72 hr before performing the assays. After the seeds were set at the center of the arena, the number of seeds carried to the nest by the ants was recorded every 15 min for 90 min.

### 2.4 Bioassay using the dummy seeds made of filter paper

We performed a bioassay using dummy seeds made of a square bit of filter paper (2 mm × 2 mm). Although the size of dummy seeds was larger than the real seeds (Figure 1), the size is the minimum to track the outcome of the dummy seeds. The dummy seeds were numerically numbered with a carbon pencil, enabling to refer the treatment conditions for each assay. The dummy seeds were soaked with the volatile compounds of *M*. *humile* seeds (Table 1). These compounds were purchased from Wako Pure Chemical Industries (Osaka, Japan) or Tokyo Kasei (Tokyo, Japan) with >98.0% purity.

**TABLE 1:** Odor components of *M*. *humile* seeds identified by GC/MS analysis. Six identified components and their mixture were used in our bioassays

Peak No.	Components	Molecular formula	Molecular weight	Retention Index	Diagnostic ions (*m/z*)
1	Isobutyl alcohol	C_4_H_10_O	74.1	1,073	33, 43, 55, 74
2	Unidentified	—	—	1,186	43, 55, 70
3	Isoamyl alcohol	C_5_H_12_O	88.2	1,208	42, 55, 70
4	Isoamyl hexanoate	C_11_H_22_O_2_	186.3	1,480	43, 70, 99, 117
5	Linalool	C_10_H_18_O	154.2	1,573	41, 43, 55, 71, 93, 121, 136, 154
6	Isobutyric acid	C_4_H_8_O_2_	88.1	1,588	43, 73, 88
7	α‐Terpineol 98.0%	C_10_H_18_O	154.2	1,725	43, 59, 81, 93, 121, 136
8	Unidentified	—	—	1,852	41, 60, 69, 93, 123

Note

**FIGURE 3:**
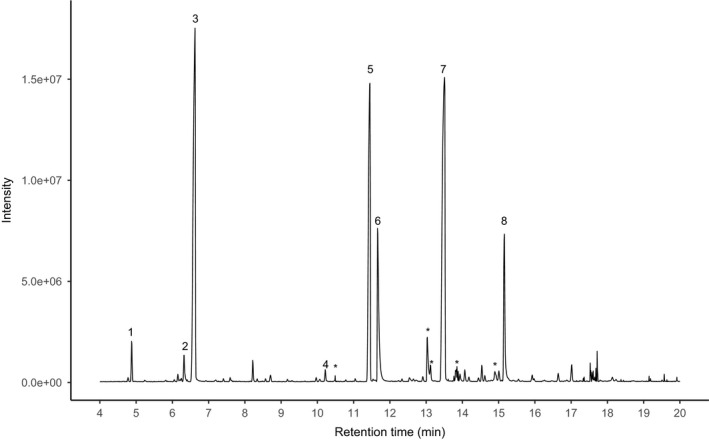
Total ion chromatogram of SPME collection of volatiles from *M*. *humile* seeds. Peak identification: (1) isobutyl alcohol; (2) unknown; (3) isoamyl alcohol; (4) isoamyl hexanoate; (5) linalool; (6) isobutyric acid; (7) α‐terpineol; (8) unknown. Peaks marked with an asterisk are artifacts also detected from blank analyses

Peak numbers correspond to those in Figure 3

In this dummy‐seed assay, 20 pieces of filter paper were set for each assay. The odor treatment in this assay consisted of eight conditions: each of the six odors identified alone (six conditions), a mixture of equivalent amounts of the six odors (one condition), and the control condition (ethanol alone). Each odor component was diluted to a final concentration of 100 ng/µl using 99.5% ethanol as a solvent. Twenty pieces of filter paper were soaked in 20 µl odor solution (1 µl for each filter paper). Each of the eight odor treatments was replicated six times using six different colonies, and each colony experienced each treatment only once. The colonies were starved (only given water) for 72 hr before performing the assays. During each turn of assay (72 hr), ant colony was supplied nothing other than the dummy seeds. The interval between assay turns for each colony was about a week in minimum.

The order of the eight odor treatments that were served to each colony was randomized using the “sample” function of “base” package of R (R core team, 2018). Dummy seeds were placed at the center of the arena, and the number of dummy seeds carried to the nest by the ants was recorded every 15 min for 90 min. After 72 hr from setting the dummy seeds, the number of dummy seeds inside the nest and discarded outside the nest was recorded. For discarded dummy seeds, we also recorded their destination and numerical number on the dummy seeds for about two weeks–two months for each ant colony. We also recorded where the ant corpses were disposed outside the nest.

### 2.5 Statistical analysis

All analyses were executed using R 4.0.3 for Mac OSX. The package “tidyverse” was used for data shaping and arrangement. The factors affecting the number of seeds (or dummy seeds) carried by ants were analyzed using a generalized linear model (GLM) and likelihood‐ratio test (“ANOVA” function from “car” package). We used the binomial distribution with the function “glm.” In this analysis, the odor conditions and the colony identity were the explanatory variables. Chi‐square test were performed to examine whether the destination of the dummy seeds was biased among the four sections of the foraging, the empty area at the center of the field, and inside the nest arena.

## 3 RESULTS

### 3.1 Volatile chemical composition of *M. humile* seeds

Chemical analyses of seed volatiles revealed the presence of several volatile compounds. By comparing the results with blank analyses, we found eight volatile compounds that were emitted from *M*. *humile* seeds with pulps (Figure 3). Among the eight peaks, six were identified as isobutyl alcohol, isoamyl alcohol, isoamyl hexanoate, linalool, isobutyric acid, and α‐terpineol (Table 1). Peaks 2 and 8 could not be identified from the obtained mass spectra.

### 3.2 Bioassay using *M. humile* seeds

**FIGURE 4:**
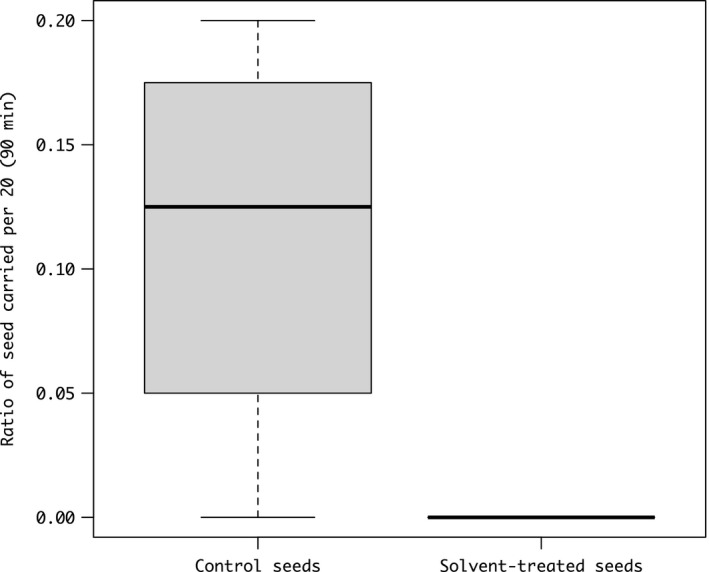
Ratio per 20 seeds of *Monotropastrum humile* carried per assay of 90 min by the ants. Twenty seeds (solvent‐treated or untreated) were placed per assay. In the solvent‐treated seeds, odor components were extracted with ethanol, whereas the untreated seeds were fresh seeds without extractions (colony replicates = 4)

The ants carried away fresh *M*. *humile* seeds in 90 min. Nine of the 80 untreated seeds were carried to the nest (mean 2.25 ± 1.71 *SD* per assay), although none of the solvent‐treated seeds were carried to the nest (Figure 4). We could not track the seed after they were carried although we did not find any seeds within the foraging arena after the assay. GLM analysis and likelihood‐ratio test revealed that untreated seeds were carried away significantly more than solvent‐treated seeds (odor of seeds, χ^2^ = 13.27, *df* = 1, *p* < .001) and not affected by colony identity (χ^2^ = 6.34, *df* = 3, *p* = .0959).

### 3.3 Bioassay using filter paper as dummy seed

**FIGURE 5:**
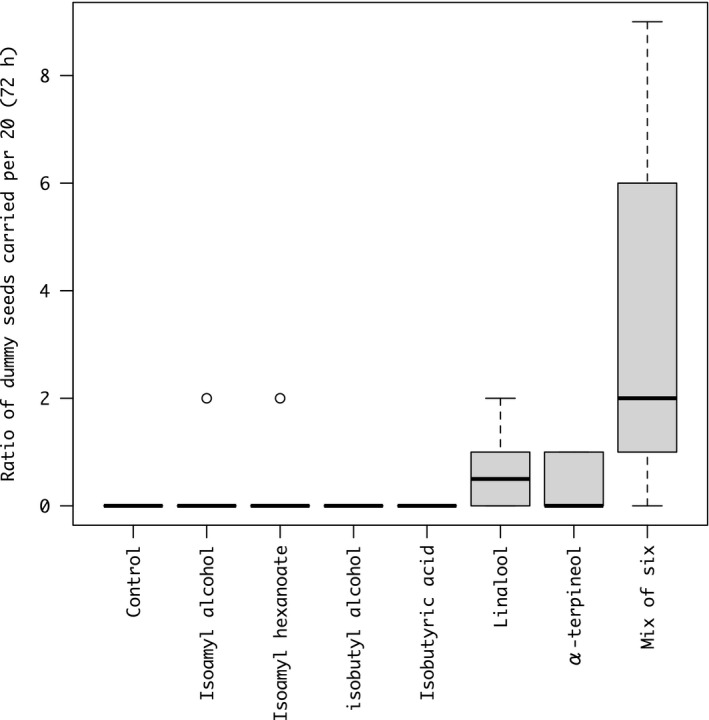
Ratio per 20 dummy seeds carried by the ants in 72‐hr observation per assay. The dummy seeds were coated with each of six seed volatile components emitted from *M*. *humile* alone, or a mixture containing all six components, or solvent‐treated control (colony replicates = 6)

Ants did not carry the dummy seeds treated with odor components to the nest in the observed 90 min but did in 72 hr. A total of 30 out of 840 odor‐treated dummy seeds were carried to the nest (mean 0.58 ± 1.51 *SD* per assay). Chemical treatments on the dummy seed significantly affect the dummy‐seed removal (χ^2^ = 63.54, *df* = 7, *p* < .001) and not affected by colony identity (χ^2^ = 9.54, *df* = 1, *p* = .089). Among each odor treatment, the dummy seeds treated with six‐odor mixture treatment were carried most to the nest (Figure 5).

**FIGURE 6:**
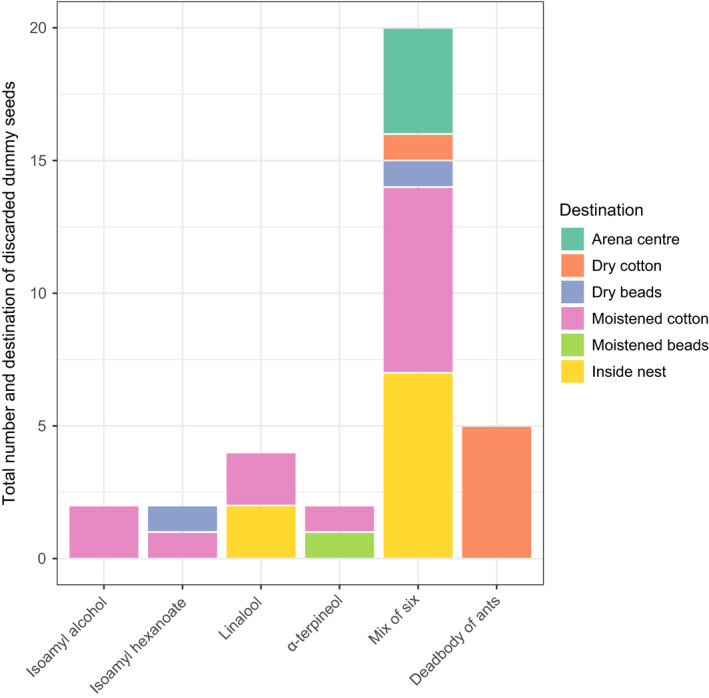
Direction of the discarded dummy seeds. Discard directions were four sections of arena (each filled with moist and dry cotton, moist and dry glass beads), arena center, and inside the nest. “Deadbodies” indicate the discards of dead ants outside the nest

Of the 30 dummy seeds collected by the ants, 17 were discarded (Figure 6). The 13 dummy seeds not discarded were left inside the nests. A chi‐square test was performed on the following six categories: the four sections of the foraging field, the empty area at the center of the field, and inside the nest. Based on this analysis, there was a significant bias toward discarding a specific location (χ^2^ = 20, *df* = 5, *p* < .001). Most of the seeds were discarded on moistened glass beads excluding left inside the nest. Five of ant corpses were disposed to the arena of dry cotton (Figure 6).

## 4 DISCUSSION

### 4.1 Dispersal of elaiosome‐less seeds by ants

In this study, we first verified that *M*. *humile* seeds without elaiosomes indeed attracted ants, whereas no solvent‐treated seeds were collected. Because solvent treatment removes various chemical compounds from the seeds, such as fatty acids, amino acids, and volatile chemical, the results suggest the chemical signaling is important factor mediating the seed dispersal by ants. We also found that by using dummy seeds coated with the odor components of *M*. *humile* seeds, dummy seeds treated with a mixture of six major components were carried to the nest by the ants, and the dummy seeds treated with the solvent were not carried away. The dummy seeds coated with a single‐odor were also less carried by the ants compared with the dummy seeds coated with six‐odor components. These results indicate that volatile odor mixtures are involved in attracting ants and inducing seed‐carrying behavior. Although some real seeds were removed with 90 min, none of the dummy seeds coated with odor components were removed. Because multiple factors, such as shapes and textures, could mediate the seed dispersal by ants, our results also suggest that the combination of other factors with seed odors is important to induce the efficient seed removal in this system.

The dummy seeds were abandoned outside the nest at the moment the volatile seed odors would have been less‐concentrated or disappeared. The pulps are tightly adhered to seeds, and it is difficult to discriminate whether volatile odors are emitted from fruits or seeds in our analyses. If the odors were emitted from pulps, the seeds are likely to be abandoned after the outer fruity coating components are consumed, and then, it is possible that *M*. *humile* could disperse their seeds through ants even in the absence of elaiosomes. Future studies should examine the origin of volatile odors and the persistence of the emitted odor components.

The elaiosome of typical myrmecochorus seeds are known to induce seed dispersal by nonvolatile chemicals such as fatty acids and amino acids, and the role of volatile chemicals is less‐studied (Nelson et al., 2019). Volatile chemicals enable seed recognition by ants from distance and, in combination with nonvolatile chemicals, may allow for more reliable and effective seed dispersal. The contribution of seed‐derived odor mixtures for their dispersal has been reported in specific obligative ant–plant mutualism. In the pepper family plant, *Peperomia macrostachya*, a mixture of seed odors efficiently attracts the ant, *Camponotus femoratus* (Youngsteadt et al., 2008). In such an obligative seed‐dispersal system, ants actively respond to the odor of the seed and disperse the seed. In contrast, *M*. *humile* plant is not completely dependent on ants for seed dispersal, as Suetsugu (2017) has reported that many seeds have been recovered from the excretion of herbivorous insects. Hence, ants are thought to participate in the seed dispersal of *M*. *humile,* in addition to herbivorous insects, such as cockroaches and camel crickets (Suetsugu, 2017; Uehara & Sugiura, 2017). Since the number of seeds carried away in our assay was relatively small, myrmecochory of the species could be just one aspect of their zoochory. In such facultative, multispecies seed‐dispersal interactions, the species‐specific signaling between ants and plants might be less likely, and more flexible communication will mediate the facultative seed‐dispersal system. The associative learning of odors with food rewards could mediate such a flexible communication and allows ant colonies to choose which seeds to be carried based on the colony nutritional needs. The plant seeds are often presented to the disperser along with fruits that have nutritional values for ants, as in the case of *M*. *humile* seeds used in this study. Ants are known to learn quickly to associate odor and reward (Dupuy et al., 2006), and such learning plays an important role in recognizing mutualistic partners in ants (Hojo et al., 2014).

Among the six main odor components in the present study, linalool and α‐terpineol were also identified as floral scents of *M*. *humile*, but the other four components were not identified (Kubo & Ono, 2014). These results indicate that *M*. *humile* seeds attract ants via their seed or fruit‐specific odor mixture. By learning to associate such specific odor mixtures with their reward, ants may be able to effectively find seeds and disperse them. Future experiments focusing on learning by seed dispersers will contribute to deepen our understanding about the signaling in facultative seed‐dispersal interactions.

### 4.2 Implication by tracking of dummy seeds

Seed dispersal by ants could be beneficial for achlorophyllous species. Although we could not track where the real seeds were discarded because of their small size, the author M. Y. observed that some of the real seeds carried into the nests were left in a corner of the nest (data not shown). Thus, the ant nest could be one of the seed destinations. Although granivory inside the nest by ants cannot be rejected, dummy seeds coated with mixed odors were carried into the nest and subsequently a part of them were discarded outside of the nest by the ants. Because *N*. *flavipes* nests are located under litter or on rotting trees (Kallal & LaPolla, 2012), it might be possible that seeds left in the nest can also be considered dispersed if they could avoid the granivory from ants. Although we did not copy the true conditions of forest floors, the target of discards was biased, and the dummy seeds were discarded mostly onto the water‐moistened glass beads. In the ant species *Myrmica rubra*, it is known that workers discard inert items and corpses in different destinations (Diez et al., 2012). In our study, it was also observed that ant corpses were disposed of in cotton wool compartments, where the dummy seeds were not discarded. These results suggest that the ants might differentiate among discard destinations depending on the material (e.g., plant‐ or animal‐originated) and that the target of ants for discarded seeds may be areas like the water‐moistened sections in our experiments. Because mycorrhizal fungi of *Russula* species, the hosts of *M*. *humile,* generally inhabit the moistened area of the forest floor (Courty et al., 2008; Rachel, 2004), it is possible that discarded area (moist soil near the surface) is close to their hosts and thus suitable for germination of *M*. *humile* seeds. Further experiments to verify the germination rate of *M*. *humile* seeds at the discarded area are needed to confirm the directed seed dispersal by ants in *M*. *humile*.

## 5 CONCLUSION AND CAVEATS

We verified that seeds of achlorophyllous and myco‐heterotrophic *M*. *humile* were dispersed by ants although they have no elaiosomes. We found that plant‐specific volatile mixtures are involved in the attraction of ants and subsequent seed‐carrying behavior. A part of the dummy seeds was left in the ant nests and the other part may be discarded outside the nest directionally, although we could not evaluate the fate of real seeds. In future studies, we must track the fate of genuine seeds and investigate the spatial distribution of the plants and ant nests to validate the directed seed dispersal of the achlorophyllous plant *M*. *humile*. An expected method to perform this bioassay is to copy various conditions of the forest floors and examine the directed dispersal of seeds by ants in detail.

## CONFLICT OF INTEREST

There are no conflicts of interest to declare.

## AUTHOR CONTRIBUTIONS

**Mikihisa Yamada:** Data curation (equal); Investigation (equal); Writing‐original draft (lead). **Masaru K. Hojo:** Conceptualization (supporting); Data curation (equal); Funding acquisition (supporting); Methodology (equal); Supervision (equal); Visualization (supporting). **Akio Imamura:** Data curation (equal); Investigation (equal); Methodology (equal); Project administration (lead); Supervision (lead); Writing‐review & editing (lead).

## Data Availability

All relevant data are within the paper and its Supporting Information files.
